# Complete genome sequence of *Halobacillus* sp. strain 12SKB, a carotenoid-producing bacterium isolated from Kakaban Lake, an anchialine lagoon within a raised atoll in East Kalimantan, Indonesia

**DOI:** 10.1128/mra.01139-25

**Published:** 2026-01-30

**Authors:** Andri Frediansyah, Hayatun Nufus, Imam Bagus Nugroho, Harald Gross

**Affiliations:** 1 Research Center for Food Technology and Processing, National Research and Innovation Agency (BRIN) 599846, Wonosari, Daerah Istimewa Yogyakarta, Indonesia; 2 Department of Agro-industrial Technology, Faculty of Agricultural Technology, Universitas Gadjah Mada 59166, Yogyakarta, Daerah Istimewa Yogyakarta, Indonesia; 3 Department of Pharmaceutical Biology, Pharmaceutical Institute, University of Tübingen 9188, Tübingen, Germany; University of Maryland School of Medicine, Baltimore, Maryland, USA

**Keywords:** *Halobacillus*, halophilic, carotenoids

## Abstract

*Halobacillus* sp. 12SKB was isolated from a unique Indonesian brackish water lake ecosystem, and its genome sequence was determined. Here, we report its 4.1-Mb genome, which gives insight into the taxonomic position and the secondary metabolite production capacity and reveals genes putatively responsible for its halophilic properties and orange occurrence.

## ANNOUNCEMENT

As part of our ongoing efforts to explore extremophilic bacteria, strain 12SKB was isolated from sediment of the Kakaban Lake, Indonesia (2.1403°N, 118.5091°E). Isolation was performed using the serial dilution plating method on Zobell marine agar (1, 2). By whole-genome sequencing, we aimed to clarify its taxonomic position, to reveal the genetic background of its properties, and to discover new secondary metabolites by genome mining (3).

For gDNA extraction, a 1 mL bacterial culture was grown for 24 h in Zobell marine broth (1, 2) at 25°C and 150 rpm. Genomic DNA from strain 12SKB was extracted using the Wizard gDNA Purification Kit (Promega), with a modification that included lysozyme treatment. The harvested cell pellet was resuspended in 480 µL of 50 mM EDTA; 120 µL lysozyme was added; and the mixture was incubated at 120 µL at 37°C for 30–60 min. Subsequent steps followed the manufacturer’s protocol. DNA purity and concentration were assessed using a NanoDrop spectrophotometer and quantified with a Qubit 3 fluorometer, yielding a final concentration of 91.2 ng/µL. A ligation-based library was prepared using the SQK-NBD114-24 Ligation Kit (Oxford Nanopore Technologies) from high-molecular-weight gDNA (~4.07 Mb genome). The DNA was neither mechanically sheared nor size-selected, as its quality and fragment length were sufficient for sequencing. DNA integrity was confirmed using an Agilent Femto Pulse System. Libraries were loaded on a FLO-PRO114M flow cell and sequenced with MinKNOW (v24.06.10) using the high-accuracy basecalling model (v4.3.0) with a minimum Q-score cutoff of 9. Raw FASTQ files were generated at a frequency of one file every 10 min. Adapter and barcode trimming was not enabled during basecalling (Trim barcodes: Off). Therefore, adapter sequences and barcodes were removed during downstream processing using Guppy (v3.2.6). Quality control of raw reads included filtering out reads with quality scores below Q9. Error correction was performed using Medaka v2.0.0 to improve base accuracy prior to genome assembly. *De novo* genome assembly was conducted using a workflow on the Galaxy Europe platform (4). Raw Nanopore FASTQ reads were uploaded to the Galaxy server and merged into a single data set using Galaxy’s ‘Concatenate data sets’ tool, which was then assembled with Flye v2.9.6 (5). Consensus contigs were evaluated using Bandage v0.9.0 (6), QUAST 5.3.0 (7), and BUSCO v5.8.2 (8) to assess assembly quality and completeness. Annotation was performed with the National Center for Biotechnology Information (NCBI)-Prokaryotic Genome Annotation Pipeline (PGAP) v6.10 (9, 10), and ribosomal RNA genes were identified using Barrnap v0.9. For all software tools, default parameters were used, unless otherwise specified. Table 1 summarizes the genomic information.

**TABLE 1:** Sequencing metrics for *Halobacillus* sp. 12SKB and bioinformatic analysis results

Statistic or characteristic	Data for *H*. sp. 12SKB
Sequencing	
No. of reads	2,055,825
Subread *N*_50_ [bp]	1,927.0
Coverage [×]	619
Assembly	
No. of contigs	2
Contig 1 (plasmid) size [bp]	3,090
Contig 2 (chromosome) size [bp]^*a*^	4,069,864
GC content [%] (chromosome/plasmid)	43.5/43.17
Estimated genome completeness [%]^*b*^	100
No. of protein coding genes (chromosome/plasmid)	3,966/6
rRNAs	24
tRNAs	59
ncRNAs	4
WGS-based screening for biosynthetic gene clusters^*c*^	
Terpenoids	3 (one thereof coding for carotenoids)
Ectoine	1
Type III polyketide	1
Ribosomal peptide	1
Siderophores	2
Betalactone	1
WGS-based taxonomy	
dDDH value *d*_4_ [%]^*d*^ (closest type strain)	37.9 (*Halobacillus faecis* NBRC 103569^T^)
ANI value [%]^*e*^ (closest type strain)	90.2 (*Halobacillus aidingensis* CGMCC 1.3703^T^)

^*a*^ The genome assembly produced a single circular contig confirmed by Bandage visualization. Overlapping ends were not trimmed, and the genome was not rotated to a specific reference start position; thus, the circular genome is presented in its assembled orientation.

^*b*^ Determined by BUSCO v5.8.2 analysis using the bacillales_odb10 dataset.

^*c*^ Biosynthetic gene clusters coding for secondary metabolites identified using antiSMASH v8.

^*d*^ dDDH: digital DNA-DNA-hybridization, determined with TYGS.

^*e*^ ANI: average nucleotide identity determined with autoMLST.

Analysis of dDDH *d*_4_ and ANI values (Table 1) showed that in both cases, the determined values are below the species delineation threshold (11–13). Thus, 12SKB represents a new candidate *Halobacillus* species. Secondary metabolism analysis using antiSMASH v8 (14) (Table 1) unveiled loci for the biosynthesis of carotenoids and ectoine, which explains its orange pigmentation and halophilic properties, respectively, and underscores its biotechnological potential (15–19).

## Data Availability

The whole-genome assembly of strain 12SKB is available at NCBI GenBank (accession number JBRFWR000000000), and raw sequencing reads have been deposited under accession number SRX30704041.
